# The diarrhetic shellfish-poisoning toxin, okadaic acid, provokes gastropathy, dysbiosis and susceptibility to bacterial infection in a non-rodent bioassay, *Galleria mellonella*

**DOI:** 10.1007/s00204-021-03132-x

**Published:** 2021-08-10

**Authors:** Helena Emery, William Traves, Andrew F. Rowley, Christopher J. Coates

**Affiliations:** Department of Biosciences, Faculty of Science and Engineering, Swansea University, Swansea, Wales, SA2 8PP UK

**Keywords:** Histopathology, 16S (V3–V4) rRNA microbiome, Immune-compromised, ﻿In vivo model, Food poisoning, Marine toxins

## Abstract

Diarrhetic shellfish-poisoning (DSP) toxins such as okadaic acid and dinophysistoxins harm the human gastrointestinal tract, and therefore, their levels are regulated to an upper limit of 160 μg per kg tissue to protect consumers. Rodents are used routinely for risk assessment and studies concerning mechanisms of toxicity, but there is a general move toward reducing and replacing vertebrates for these bioassays. We have adopted insect larvae of the wax moth *Galleria mellonella* as a surrogate toxicology model. We treated larvae with environmentally relevant doses of okadaic acid (80–400 μg/kg) via intrahaemocoelic injection or gavage to determine marine toxin-related health decline: (1) whether pre-exposure to a sub-lethal dose of toxin (80 μg/kg) enhances susceptibility to bacterial infection, or (2) alters tissue pathology and bacterial community (microbiome) composition of the midgut. A sub-lethal dose of okadaic acid (80 μg/kg) followed 24 h later by bacterial inoculation (2 × 10^5^ *Escherichia coli*) reduced larval survival levels to 47%, when compared to toxin (90%) or microbial challenge (73%) alone. Histological analysis of the midgut depicted varying levels of tissue disruption, including nuclear aberrations associated with cell death (karyorrhexis, pyknosis), loss of organ architecture, and gross epithelial displacement into the lumen. Moreover, okadaic acid presence in the midgut coincided with a shift in the resident bacterial population over time in that substantial reductions in diversity (Shannon) and richness (Chao-1) indices were observed at 240 μg toxin per kg. Okadaic acid-induced deterioration of the insect alimentary canal resembles those changes reported for rodent bioassays.

**Supplementary Information:**

The online version contains supplementary material available at 10.1007/s00204-021-03132-x.

## Introduction

The lipophilic toxin okadaic acid and structurally similar dinophysistoxins are produced by several marine dinoflagellates, notably species of *Prorocentrum* and *Dinophysis,* and cause diarrhetic shellfish poisoning (DSP) in humans (Tachibana et al. 1981; Yasumoto et al. 1985; Trainer et al. 2013). DSP toxins are the most common output from harmful algal blooms in European, South American, and Asian waters, thereby representing the leading cause of aquaculture harvesting bans and site closures in Europe (Reguera et al. 2014; Dhanji-Rapkova et al. 2018; Bresnan et al. 2021). After consuming contaminated shellfish tissues, usually from phytoplankton filtering bivalves, humans can develop nausea, vomiting, incapacitating diarrhoea, abdominal cramps, and, in some cases, chills and fever within 0.5–5 h. The structural integrity of DSP toxins remain intact after cooking and freeze-thawing (McCarron et al. 2008; Reboreda et al. 2010), and their presence in shellfish flesh does not appear to alter the organoleptic profile (Valdiglesias et al. 2013). Okadaic acid inhibits serine/threonine phosphatases (PP1, PP2A; Bialojan and Takai 1988), disrupting cellular homeostasis due to hyperphosphorylation and leading to downstream dysregulation of the gastrointestinal mucosa. The broad symptomology of okadaic acid-induced poisoning cannot be attributed to phosphatases alone and likely involves the enteric nervous system (e.g., neuropeptide Y; Louzao et al. 2015). Recent experiments carried out on Swiss female mice suggest that serotonin (5-hydroxytryptamine) levels are a contributing factor, as 0.1–10 mg/kg of cyproheptadine (a serotonin antagonist) delayed or prevented diarrhoea entirely (Louzao et al. 2021).

Beyond the GI tract, organs such as the kidneys, liver, and placenta can be affected, with okadaic acid demonstrating mutagenic, genotoxic, carcinogenic, and neurotoxic properties (Matias and Creppy 1996; reviewed by Mundy 2013). Chronic exposure to this polyether toxin is linked to elevated levels of tumour promoters and increased risk of cancers, e.g., colorectal (Manerio et al. 2008; Vilariño et al. 2018; Jiménez-Cárcamo et al. 2020). Because of the diverse biological properties, distribution, and frequency of DSP toxins in the marine environment, their levels are regulated across Europe—with an upper limit of 160 μg [toxin equivalents] per kg [shellfish tissue]—to protect consumers (O’Mahony 2018). The acute, subacute and chronic, low-dose immunotoxicological effects of shellfish-poisoning toxins (including DSPs) are poorly defined and understudied in humans, as are the detrimental impact they have across marine food webs, including commercial finfish and shellfish (Coates and Söderhäll 2020; Corriere et al. 2020, reviewed by Turner et al. 2021).

For many years, small vertebrates (e.g., rodents, zebrafish) and invertebrates (e.g., fruit flies, nematodes) have been used as models/proxies for humans across the biological and biomedical sciences including the study of genetics, senescence, host–pathogen interactions, and antibiotic development. Regarding invertebrates, their use brings obvious financial, time, and ethical advantages, however, those organisms mentioned above are rather small—making precise dosing challenging—despite their high-throughput capacity (Emery et al. 2019, 2021). Larvae of the greater wax moth (*Galleria mellonella*) offer combined benefits: low cost of maintenance, accurate dose administration via several routes (topical, intrahaemocoelic injection, gavage), can be used at human relevant 37 °C, and key cellular immune functions (e.g., pathogen recognition, internalisation, and destruction) mirror aspects of vertebrate innate immunity (Browne et al. 2013; Lim et al. 2018; Lange et al. 2018, 2019). For these reasons, the popularity of *G. mellonella* continues to increase as an experimental alternative for screening microbial virulence and pathogenicity, efficacy of antimicrobials, biocontrol agents, and to discriminate between toxic and non-toxic substances (Alghoribi et al. 2014; Champion et al. 2016; Allegra et al. 2018; de Barros et al. 2019; Grizanova et al. 2019, 2021; Piatek et al. 2020; Emery et al. 2021; Moya-Andérico et al. 2021; Krachler et al. 2021). Recently, we reported on the effects of *G. mellonella* exposure to environmentally relevant doses of okadaic acid (~ 80–400 μg/kg) and recorded cytotoxic (immune cell death) and gastrotoxic (REDOX imbalance) effects similar to known toxicological endpoints for the established mouse bioassay (Coates et al. 2019). This study demonstrated the potential use of *G. mellonella* for assessing the risk of marine toxins in vivo, or complementing in vitro cell-line-based assays (e.g., Caco-2) prior to vertebrate use.

In the present study, our overall aim was to define okadaic acid-related health decline and pathophysiological condition in the aspirant toxicology model, *G. mellonella*. First, we determined whether pre-exposure of larvae to a low dose of okadaic acid (80 μg/kg), followed 24 h later by 2 × 10^5^ CFUs of a common laboratory bacterium (*Escherichia coli*), led to enhanced susceptibility to infection. Second, we used tissue histology and 16S rRNA high-throughput sequencing (V3-V4 region) to compare pathological modalities between the intoxicated (mid)gut tissues of *G. mellonella* and rodent bioassays.

## Materials and methods

### Toxin and bacterium

Okadaic acid (C_44_H_68_O_13_) was purchased from TOCRIS Biosciences (UK; Cat. No. 1136). Solutions of okadaic acid were prepared in 0.2 μm filter-sterilised phosphate-buffered saline (PBS) pH 7.4 containing 5% dimethyl sulfoxide (DMSO; v/v). All remaining reagents, unless stated otherwise, were sourced from MERCK (formerly Sigma-Aldrich, Dorset, UK) in their purest form available. The bacterial target *Escherichia coli* K12 (LZB 035) was sourced from Blades Biological Ltd (Kent, UK) on nutrient agar slopes.

### *Galleria mellonella* intoxication and infection trials

Final instar larvae (*G. mellonella*) were purchased from Live Foods Direct Ltd (Sheffield, UK) and stored at 14–15 °C in the dark for no more than 5 days prior to use. Healthy larvae weighing 250–320 mg each—showing no signs of pupation, melanisation, or infection—were administered with okadaic acid either by intrahaemocoelic injection (INJ) through the last left (ventral) pro-leg or orally (force-fed, FF) using a sterile (disposable) 27-gauge hypodermic needle across the concentration range 80–400 μg/kg. Larvae were chilled on ice for no more than 2 min immediately prior to inoculation. The negative control consisted of PBS pH 7.4 containing 5% (v/v) DMSO. The volume was standardised to 10 μl per insect regardless of the inoculation route (gavage/injection) or the contents of the inoculum (chemical/microbial). Post-intoxication, larvae were incubated in the dark at 30 °C. The health of each larva was assessed over a 72-h period using an abridged scoring system developed by Loh et al. (2013) for this insect species [melanisation (0–4), activity (0–3)]. Levels of melanisation are used as indicators of infection/immunity/stress in insects (Whitten and Coates 2017)—detrimental accumulation of the black/brown pigment scores 0 (i.e., highly melanised, compromised larvae), whereas an integument free of pigmentation scores 4 (i.e., healthy, naive larvae).

Single colonies of bacterial targets, *E. coli* (Gram-negative), were selected from nutrient agar and sub-cultured into nutrient broth and grown overnight at 30 °C (200 rpm). Bacteria were handled using standard axenic practices. Once bacterial suspensions reached an optical density (OD_600_) value of 1 (VWR 1200 spectrophotometer), cells were centrifuged at 1000 × g for 5 min, washed once in PBS pH7.4, and then diluted to the required doses. The numbers (dose) of bacterial colony-forming units (CFUs) used in infection studies were 2 × 10^5^, 5 × 10^5^, 1 × 10^6^, and 2 × 10^6^ per insect.

In the final set of toxicity studies, insects were pre-exposed to the lowest dose of okadaic acid via force feeding (80 μg/kg), followed 24 h later by *E. coli* (2 × 10^5^) challenge directly into the haemocoel. Controls consisted of the following inoculation combinations, PBS (FF) + PBS (INJ), PBS (FF) + bacteria (INJ), and OA (FF) + PBS (INJ). Larvae were surface sterilised with 70% ethanol prior to any microbial inoculation to avoid potential contamination from the integument.

### Tissue histology of larval midgut

Larvae were inoculated orally with OA (80 and 240 μg/kg) or PBS (+ 5% DMSO)—doses determined previously by Coates et al. (2019)—and sacrificed at 4, 24, and 48 h by fixation in 10% formalin overnight at room temperature. Fixed larvae were cut into three parts; head, middle, and rear in preparation for paraffin wax embedding. Samples were dehydrated in 70%, 80%, and 90% ethanol each for 1 h followed by three 1-h 100% ethanol washes. Dehydrated samples were washed in Histoclear twice for 1 h to remove any remaining fixative, followed by 50% Histoclear: 50% paraffin wax for 1 h. Wax blocks containing insect tissues were trimmed manually to minimise cutting and staining areas. Trimmed blocks were sliced into 5–7 μm sections using a microtome, loaded onto glass slides with water, and dried on a heating block to allow the wax ribbon to fully expand and stick to the surface of the glass slide. Slides were staind with haematoxylin and eosin as described by Emery et al. (2019).

### Gut microbiome analysis of okadaic acid-intoxicated larvae

Randomly selected larvae (*n* = 3 per treatment, per time point, *n* = 27 overall) that were force-fed okadaic acid (80 or 240 μg/larva) or untreated (0 μg/larva) were chilled on ice and dissected at 4, 24, and 48 h. Gut tissues (mouth to anus) were removed carefully from each larva, weighed, snap frozen in liquid nitrogen, and stored at − 80 °C before genomic DNA extraction using a Qiagen DNeasy Blood and Tissue Kit (Cat. No. 69504; Qiagen, Hilden, Germany) by following the manufacturer’s recommended protocol. DNA yields were quantified using the Qubit™ dsDNA High Sensitivity Assay Kit and Qubit™ Fluorometer (Invitrogen, California, USA) and standardised to 50 ± 2 ng/µl prior to sequencing.

Eurofins Genomics (Germany) carried out the high-throughput sequencing using their established INVIEW Microbiome Profiling 3.0 service – performed on an Illumina MiSeq platform targeting the V3–V4 hypervariable16S ribosomal RNA region (2 × 250 −300 bp) with the following oligonucleotide primers: Forward: TACGGGAGGCAGCAG (Turner et al. 1999) Reverse: CCAGGGTATCTAATCC (Kisand et al. 2002). Amplicon generation, adapter addition, quality control, size selection, pooling, demultiplexing, removal of primers, and advanced bioinformatics were performed by Eurofins (raw data processing, read merging, quality filtering, and chimera removal). De-multiplexing was carried out on all reads that were approved by the standard Illumina chastity filter. Read merging was completed using the FLASH algorithm to create a consensus sequence with the greatest quality value, considered all overlaps, and produced merged readings with the longest targeted region (Magoč and Salzberg, 2011). Where merging was not possible, the forward read was used, and merged readings were then filtered, removing reads that were too long/short. Initial microbiome profiling to remove chimeric reads was conducted using UCHIME followed by entropy decomposition analysis to form datasets of partition marker genes within operational taxonomic units (OTUs; Schloss et al. 2011; Eren et al. 2015). Each OTU was assigned using a minimum of 70% sequence identity across a minimum representative sequence of 80% using DC-Megablast (Altschul et al. 1990). OTUs were processed using QIIME software v1.9.1 (Caporaso et al. 2010) with a 97% homogeneity threshold for selection. OTUs lacking taxonomic matches were categorised as ‘unclassified’ and taxonomic units with < 0.1% reads were considered ‘other’.

OTUs at phylum and genus levels were analysed in R studio using the microbiome and vegan packages to calculate Chao-1 (richness) and Shannon (diversity) indices, and the randomised permutations test; adonis PERMANOVA (Bray–Curtis method with 999 permutations) was conducted to assess signficance between microbiomes (Cree et al. 2016). Stress and dispersion tests were performed and plotted to ensure that the model was a good fit. All samples were filtered to remove putative contaminants using any reads present in the extraction blank—OTU reads ≤ 200 were removed, leaving the remaining abundances for analysis. Replicates were merged to create average OTU values for each treatment/time point. A single sample, untreated larva at 24 h did not yield any sequence data.

### Data handling and statistical analyses

Experiments were repeated on at least three separate occasions. Results are expressed as mean ± SE (unless stated otherwise), and sample sizes can be found within the respective figure descriptors. Log-rank (Mantel–Cox) tests were used to analyse survival curves, whereas two-way ANOVAs (with Turkey’s multiple comparison tests) were used for health/damage indices in GraphPad PRIMS v7. The D’Agostino–Pearson test (Omnibus K2) was used to check data for normality, and log-transformed [log(y + 1)] when necessary. Significance values were determined when *P* ≤ 0.05. Microbiome analysis (16S region) was performed in R studio as described above.

Histology slides were visualised and singled-blind assessed as oulined in Emery et al. (2019). Briefly, a scoring index from 1 to 4 was used to indicate visible damage: (1) little change (if any), 0–2 localised tissue aberrations per slide, (2) discrete changes of 3 to 5 tissue aberrations per slide, (3) regional change representing ≥ 25% damage (the alteration is dramatic), and (4) global change of > 50% of a specific tissue type or the entire slide (gross). Images of tissue sections were adjusted for colour balance and contrast/brightness.

## Results

### Susceptibility of *Galleria mellonella* to bacteriosis in the absence and presence of okadaic acid

**Fig. 1:**
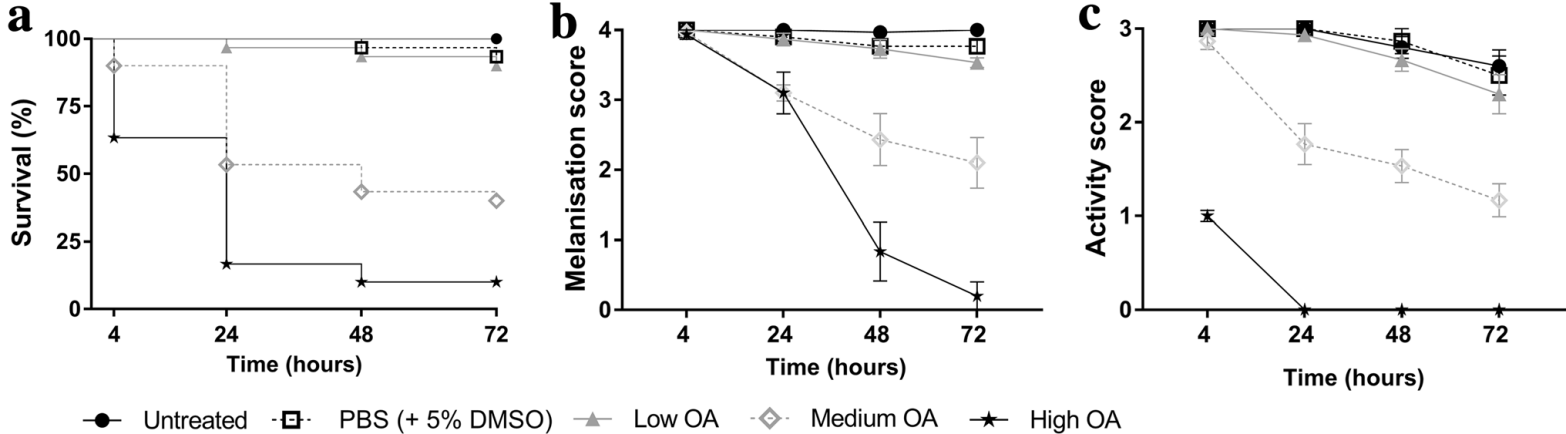
Health indices of *Galleria mellonella* force-fed okadaic acid (C_44_H_68_O_13_). Insects were exposed to 80 μg (low), 240 μg (medium), or 400 μg (high) okadaic acid and monitored for survival (**a**), melanisation (**b**), and activity (**c**) levels across the 72-h experimental period. Post-inoculation, larvae were incubated at 30 °C in the dark. Untreated larvae received no injection, and the negative control consisted of PBS (+ 5% [v/v] DMSO). Values represent the mean ± S.E. (*n* = 30 per treatment, 150 in total)

**Fig. 2:**
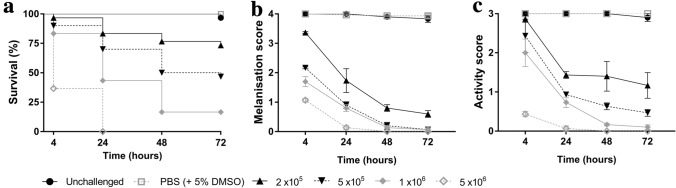
Health indices of *Galleria mellonella* infected with bacteria via intrahaemocoelic injection. Insects were exposed to *Escherichia coli* across the range, 2 × 10^5^–5 × 10^6^ CFUs, and monitored for survival (**a**), melanisation (**b**), and activity (**c**) levels during the 72-h experimental period. Post-inoculation, larvae were incubated at 30 °C in the dark. Untreated larvae received no injection, and the negative control consisted of PBS (+ 5% [v/v] DMSO). Values represent the mean ± S.E. (*n* = 30 per treatment, 180 in total)

Administering single doses of either okadaic acid via gavage (Fig. 1) or *E. coli *via intrahaemocoelic injection (Fig. 2) led to significant declines in larval health in a dose-dependent manner (*X*^2^_(4)_ = 110.7, *P* < 0.0001 [Fig. 1a] and *X*^2^_(5)_ = 157.9, *P* < 0.0001 (Fig. 2a); Table 1). Larval survival levels dropped by 10% across the 72-h experimental period when 80 μg/kg of okadaic acid was used, which is half the upper regulatory limit (i.e., 160 μg/kg) set by the European Union (EC No. 853/2004). At doses of 240 and 400 μg/kg, survival levels reduced substantially by 60% and 90%, respectively (Fig. 1a; Supplementary Table 1). At the two higher doses, there was concomitant reductions in melanisation (< 2.1 out of 4; Fig. 1b) and activity (< 1.2 out of 3; Fig. 1c) indices for the intoxicated insects. Similarly, increasing the number of *E. coli* colony-forming units (CFUs) from 2 × 10^5^ to 5 × 10^6^ led to reciprocal deteriorations in melanisation (< 0.6 out of 4; Fig. 2b) and activity (< 1.2 out of 3; Fig. 2c) indices at 72-h post-infection. In fact, no larvae survived beyond 24 h when infected with 5 × 10^6^ CFUs *E. coli*. For okadaic acid-related health decline, time and treatment accounted for ~ 7–18% and ~ 42–86% of the variation within the data, respectively. For *E. coli*-related health decline, time and treatment accounted for ~ 12–16% and ~ 76% of the variation within the data, respectively. In all cases, time, treatment, and their interaction were significant contributing factors (*P* < 0.0001; Table 1) to larval morbidity.

**Table 1:** Two-way analysis of variance (ANOVA) for *Galleria mellonella* health indices

	Source of variation	Variation (%)	Sum-of-squares	*df*	*F* value	*P* value
﻿﻿Intoxication (OA)						
Melanisation index	Time	17.5	0.314	3	27.3	< 0.0001
	Treatment	42	0.753	4	49.2	< 0.0001
	Interaction	31.9	0.571	12	12.5	< 0.0001
Activity index	Time	6.8	0.182	3	66.5	< 0.0001
	Treatment	86	2.289	4	628.6	< 0.0001
	Interaction	5.7	0.151	12	13.8	< 0.0001
Infection (*E. coli*)						
Melanisation index	Time	15.6	0.854	3	167.8	< 0.0001
	Treatment	74.8	4.1	5	483.9	< 0.0001
	Interaction	8	0.446	15	17.6	< 0.0001
Activity index	Time	11.8	0.452	3	60.8	< 0.0001
	Treatment	77	2.96	5	238.5	< 0.0001
	Interaction	8.1	0.31	15	8.3	< 0.0001
Intoxication and infection						
Melanisation index	Time	21.2	0.439	3	76.1	< 0.0001
	Treatment	47.9	0.994	4	129	< 0.0001
	Interaction	27.2	0.563	12	24.4	< 0.0001
Activity index	Time	22.6	0.151	3	50.22	< 0.0001
	Treatment	40.6	0.270	4	67.6	< 0.0001
	Interaction	30.8	0.21	12	17.1	< 0.0001

*df* degrees of freedom; *OA* okadaic acid (80 μg/kg); *Escherichia coli* (2 × 10^5^ per insect)

**Fig. 3:**
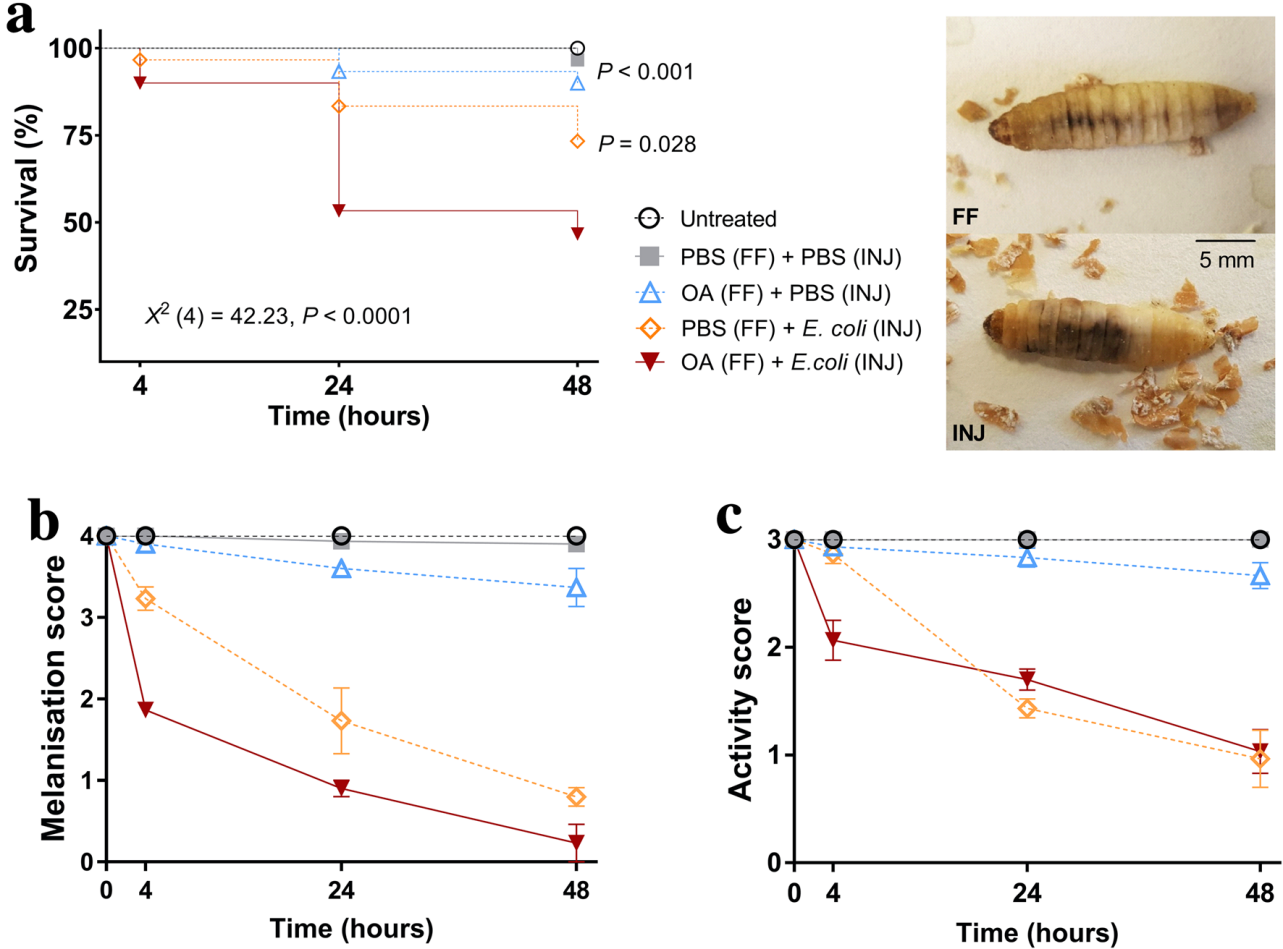
Health indices of *Galleria mellonella* challenged with a combination of okadaic acid and bacteria. Insects were pre-exposed to the low dose of okadaic acid (80 μg/kg) via gavage, followed 24 h later by an intrahaemocoelic injection of *E. coli* (2 × 10^5^ CFUs). After bacterial inoculation, survival (**a**), melanisation (**b**), and activity (**c**) levels were monitored for a further 48 h. Larvae were incubated at 30 °C in the dark. Untreated larvae received no injection, and the negative control consisted of PBS (+ 5% [v/v] DMSO) administered first by gavage, followed 24 h later by an injection into the haemocoel. Values represent the mean ± S.E. (*n* = 30 per treatment, 150 in total). Inset, images of melanisation 24 h post-force feeding (FF) an insect larva okadaic acid, or injecting bacteria directly (INJ) into the body cavity. Darkening is restricted to the alimentary canal (laterally from mouth to anus) when insects are treated orally

Pre-exposing *G. mellonella* to a low dose of okadaic acid (80 μg/kg), followed 24 h by 2 × 10^5^ CFUs of *E. coli,* caused survival levels to drop by > 53% within 48 h (Fig. 3a; *X*^2^_(4)_ = 42.23, *P* < 0.0001). Changes also corresponded with the lowest melanisation and activity indices recorded, < 0.3 out of 4 and ~ 1 out of 3, respectively (Table 1; Fig. 3b, c). Moreover, calculated hazard ratios suggest that insects receiving both toxin and bacterium were 2.7- to 6.3-fold more likely for death to occur over the same time period when compared to those receiving either challenge alone (Table 2). By replacing okadaic acid (first inoculum) or *E. coli* (second inoculum) with PBS, significantly fewer larval deaths were encountered (Table 2 for statistical outputs), with survival levels remaining above 73% (Fig. 3a). Using two inoculation routes, i.e., oral and intrahaemocoelic injection per larva, did not appear to compromise health, as indices were similar to the untreated group (*P* = 0.317; Table 2; Fig. 3a-c).

**Table 2:** Survival analyses of *Galleria mellonella* larvae inoculated with okadaic acid and bacteria alone, or in succession

	PBS (FF) + PBS (INJ)	OA (FF) + PBS (INJ)	PBS (FF) + *E. coli* (INJ)	OA (FF) + *E. coli* (INJ)
Untreated	*X*^2^ (1) = 1, *P* = 0.3173	*X*^2^ (1) = 3.106, *P* = 0.078	*X*^2^ (1) = 9.085 ***P* = 0.00526**	*X*^2^ (1) = 21.59, ***P* < 0.0001**
PBS (FF) + PBS (INJ)	–	*X*^2^ (1) = 1.09, *P* = 0.296 HR = 2.87 (95% CI, 0.39–20.72)	*X*^2^ (1) = 6.468, ***P* = 0.0110** HR = 5.7 (95% CI, 1.49–21.81)	*X*^2^ (1) = 18.74, ***P* < 0.0001** HR = 9.71 (95% CI, 3.5–26.69)
OA (FF) + PBS (INJ)	–	–	*X*^2^ (1) = 2.77, *P* = 0.95 HR = 2.83 (95% CI, 0.83–9.6)	*X*^2^ (1) = 13.37, ***P* = 0.0003** HR = 6.32 (95% CI, 2.54–15.4)
PBS (FF) + *E. coli* (INJ)	–	–	–	*X*^2^ (1) = 4.821, ***P* = 0.0281** HR = 2.72 (95% CI, 1.1–6.65)

Values represent pairwise comparisons between curves using the log-rank (Mantel–Cox) test and Mantel–Haenszel hazard ratios (*HR*). Significant differences were confirmed when *P* < 0.05, and are highlighted in bold. Okadaic acid (*OA*, 80 μg/kg) was administered via force feeding (*FF*). *Escherichia coli* (2 × 10^5^ CFUs) was administered via intrahaemocoelic injection (*INJ*)

### Effect of okadaic acid on the gut tissues of *Galleria mellonella*

**Fig. 4:**
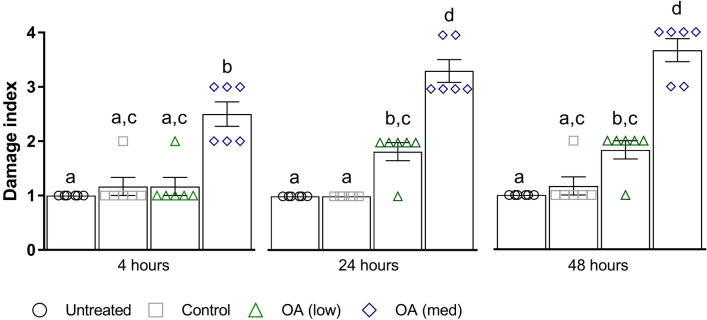
Extent of midgut tissue damage in *Galleria mellonella* after feeding okadaic acid. Okadaic acid doses represent 80 μg/kg (low) and 240 μg/kg (high). Control, or PBS + 5% (v/v) DMSO. Histology slides were single blind assessed in pairs (treatment vs control) and subsequently assigned a grade (1–4) based on damage. Grade 1 indicates little-to-no damage, whereas grade 4 represents global damage affecting > 50% of tissue. Data (mean + / − S.E.) have been compiled from assessments carried out at 4, 24, and 48 h post-inoculation. Unshared letters indicate significant differences (*P* < 0.05) determined by ANOVA and Tukey’s multiple comparison tests (*n* = 3 insects (and two technical replicates) per category per time point, 36 in total)

**Fig. 5:**
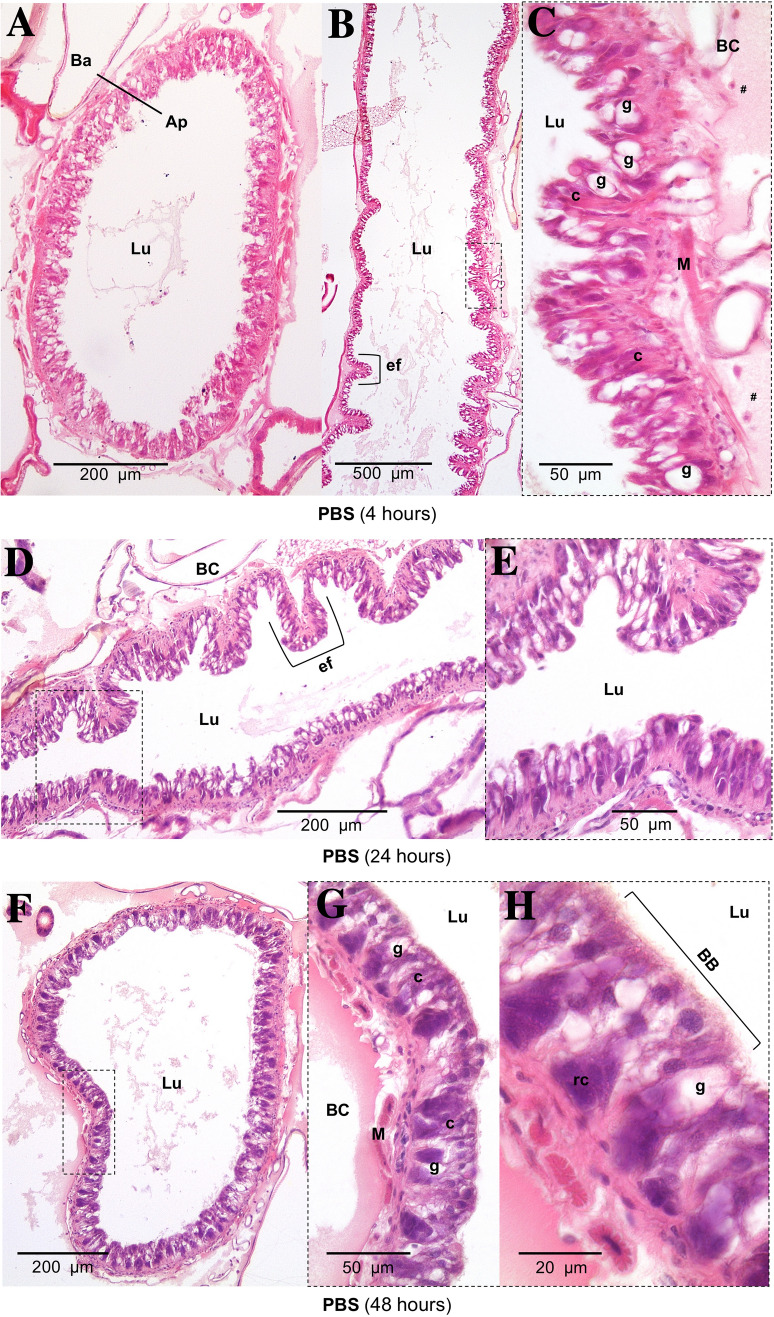
Tissue sections of the *Galleria mellonella* midgut when force-fed PBS. Photomicrographs depict transverse and longitudinal sections at 4 (panels A, B, C), 24 (panels D, E), and 48 (panels F, G, H) h post-inoculation. *Ap* apical; *Ba* basolateral; *BB* brush border; *BC* body cavity; *c* columnar (epithelial) cell; ﻿*﻿ef*, epithelia fold(s); *﻿g*, goblet cell; *﻿Lu*, lumen; *﻿M*, muscle; *﻿rc*, regeneration cell. Hashtags (#) denote haemocytes

**Fig. 6:**
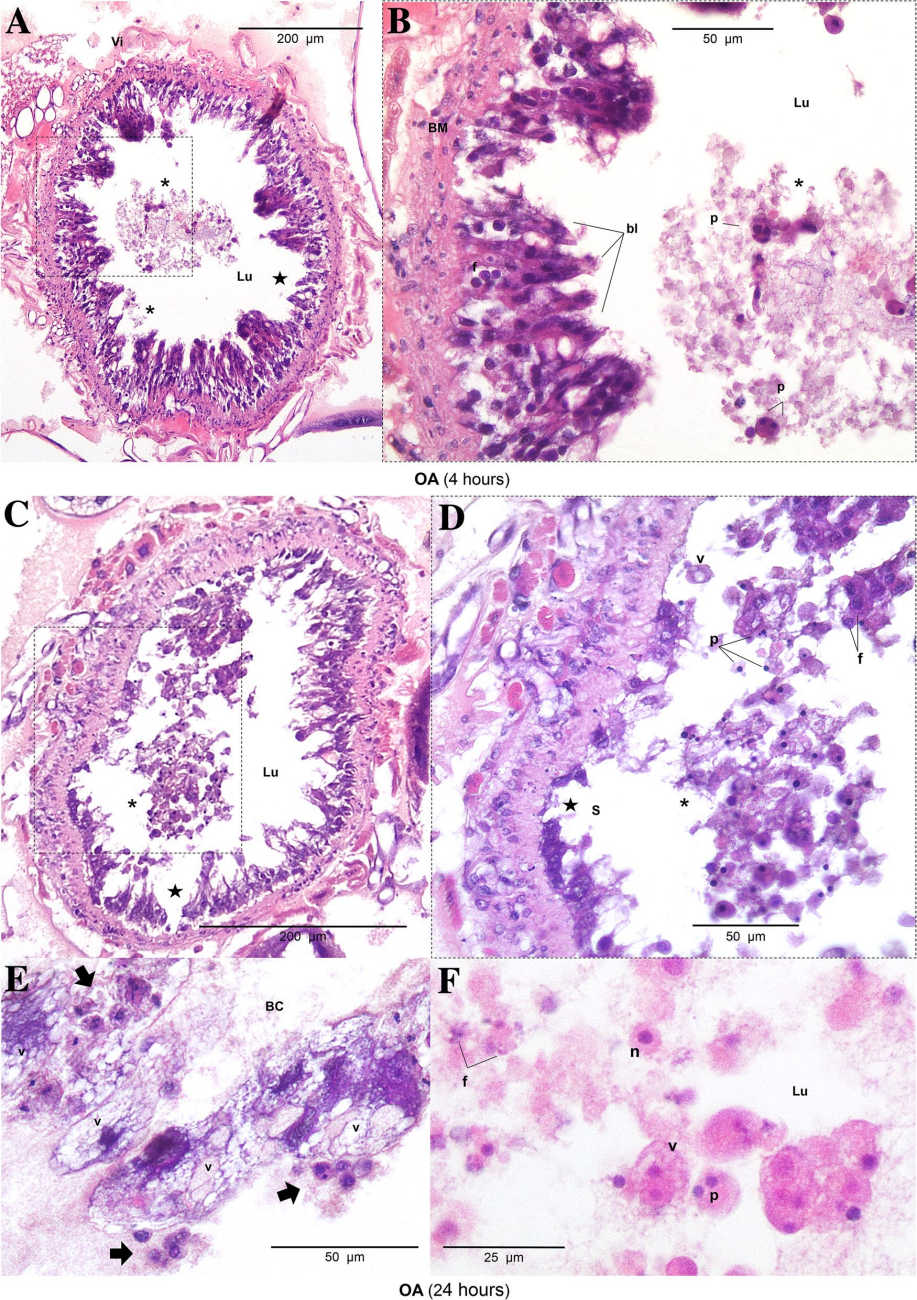

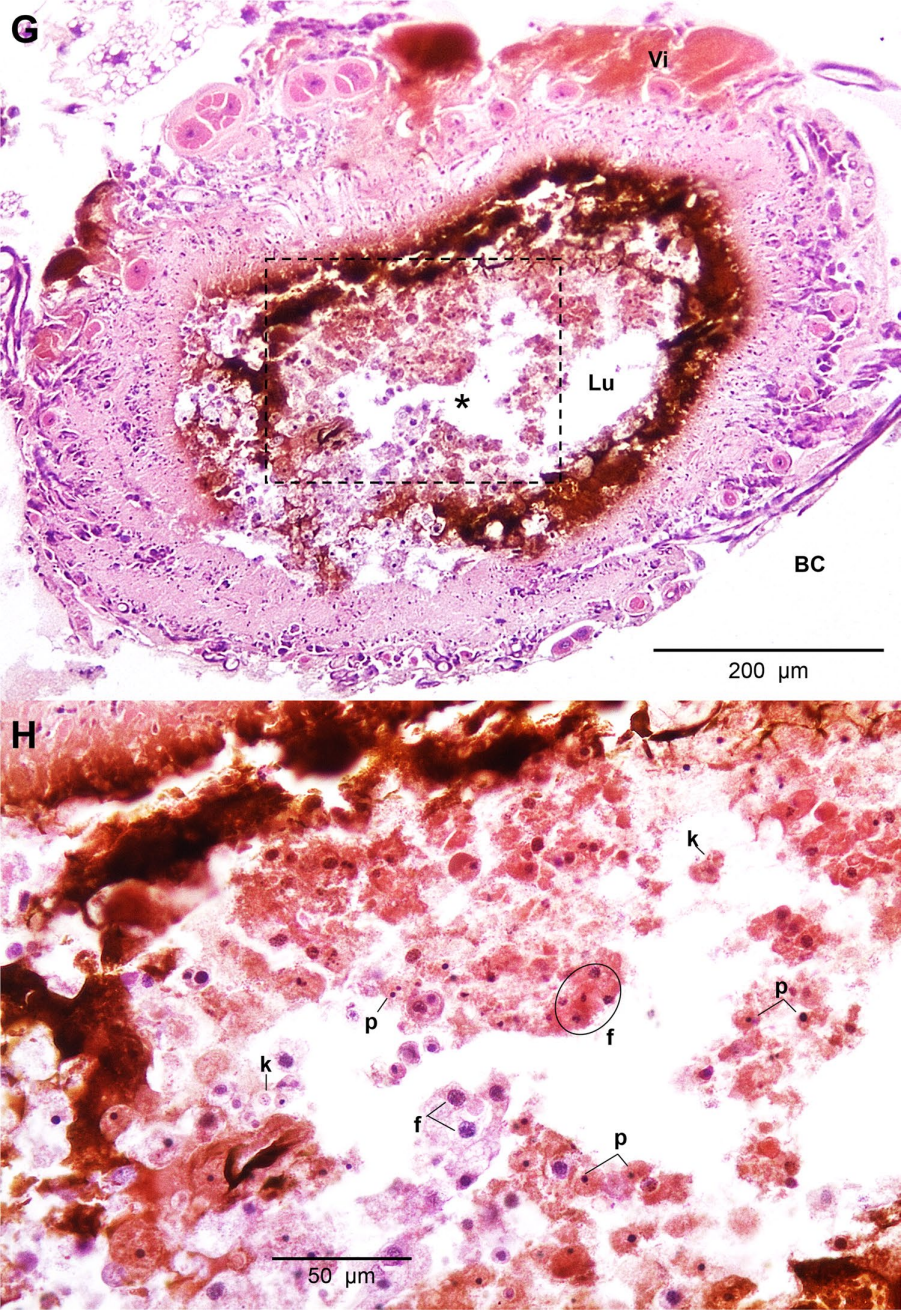
Tissue sections of the *Galleria mellonella* midgut when force-fed okadaic acid (240 μg/kg). Photomicrographs depict transverse sections at 4 (panels A, B), 24 (panels C, D, E, F), and 48 (panels G, H) h post-inoculation. *BC* body cavity; *BM*, basement membrane; *bl*, blebbing/blistering; *ef* epithelia fold(s); *f*, fragmentation (karyorrhexis); *k*, karyolysis; *Lu* lumen; *n* normal nucleus; *p* pyknosis; *v* vacuolisation; *vi* visceral muscle tissue. An asterisk (*) indicates cellular displacement. Black stars highlight areas where damage has completely removed epithelia and exposed underlying muscle. Black arrows point to compromised haemocytes. Panels G and H represent tissues at 48 h post-inoculation of okadaic acid (240 μg/larva)

**Fig. 7:**
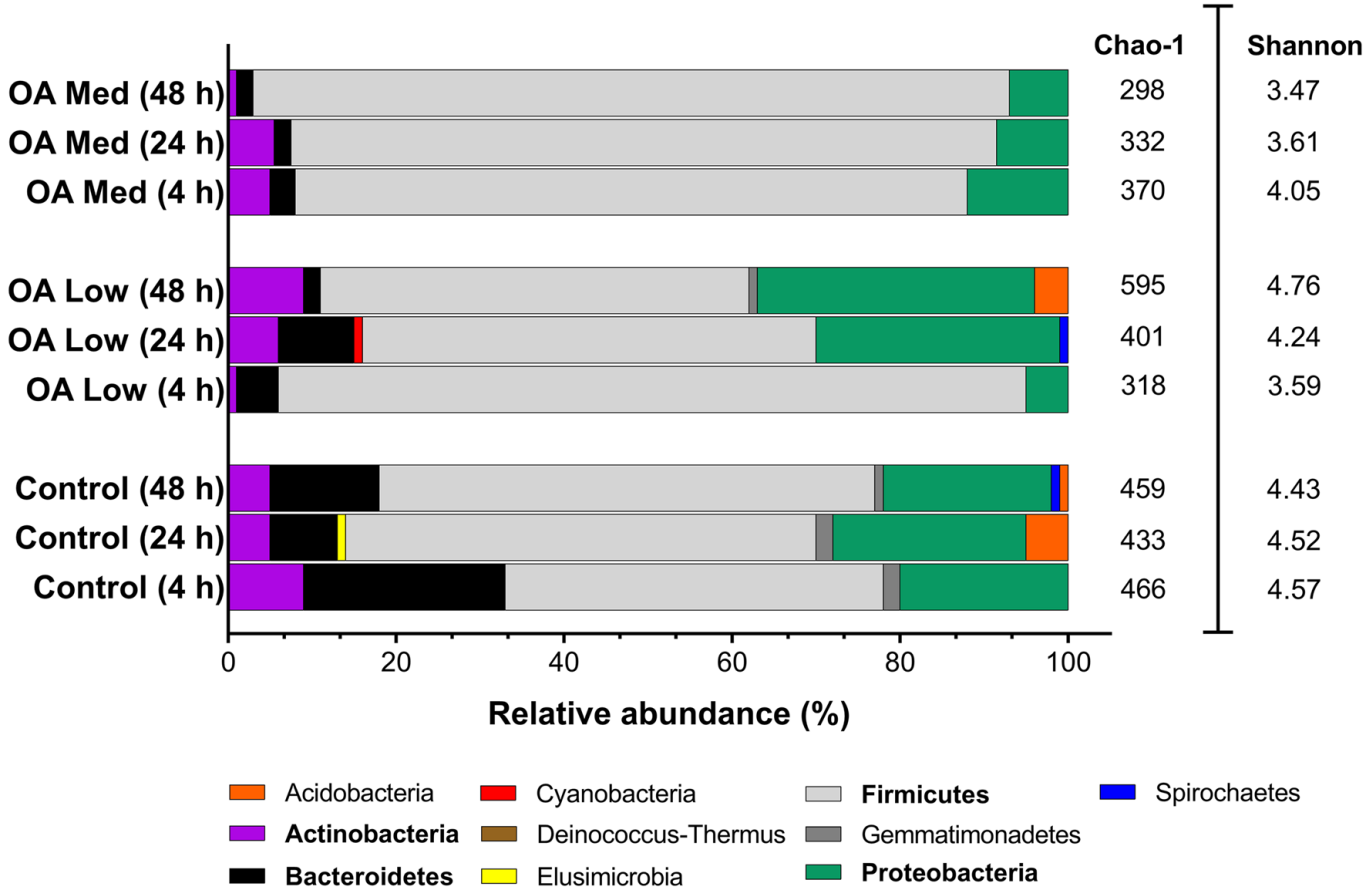
Phylum-level bacterial composition (16S rRNA V3-V4) of the gut tissues of *Galleria mellonella* during okadaic acid (C_44_H_68_O_13_) intoxication. Insects were force-fed PBS (+ 5% v/v DMSO) or okadaic acid (OA; 80 μg/kg [low], 240 μg/kg [med]) and incubated at 30 °C in the dark. At 4, 24, and 48 h post-intoxication, insects were dissected and the gut tissues were snap frozen in liquid nitrogen (*n* = 9 per treatment across time points). Inset, average Chao-1, and Shannon richness/diversity indices are placed next to the respective treatment/time point. The corresponding values for the extraction blank have been filtered out

**Fig. 8:**
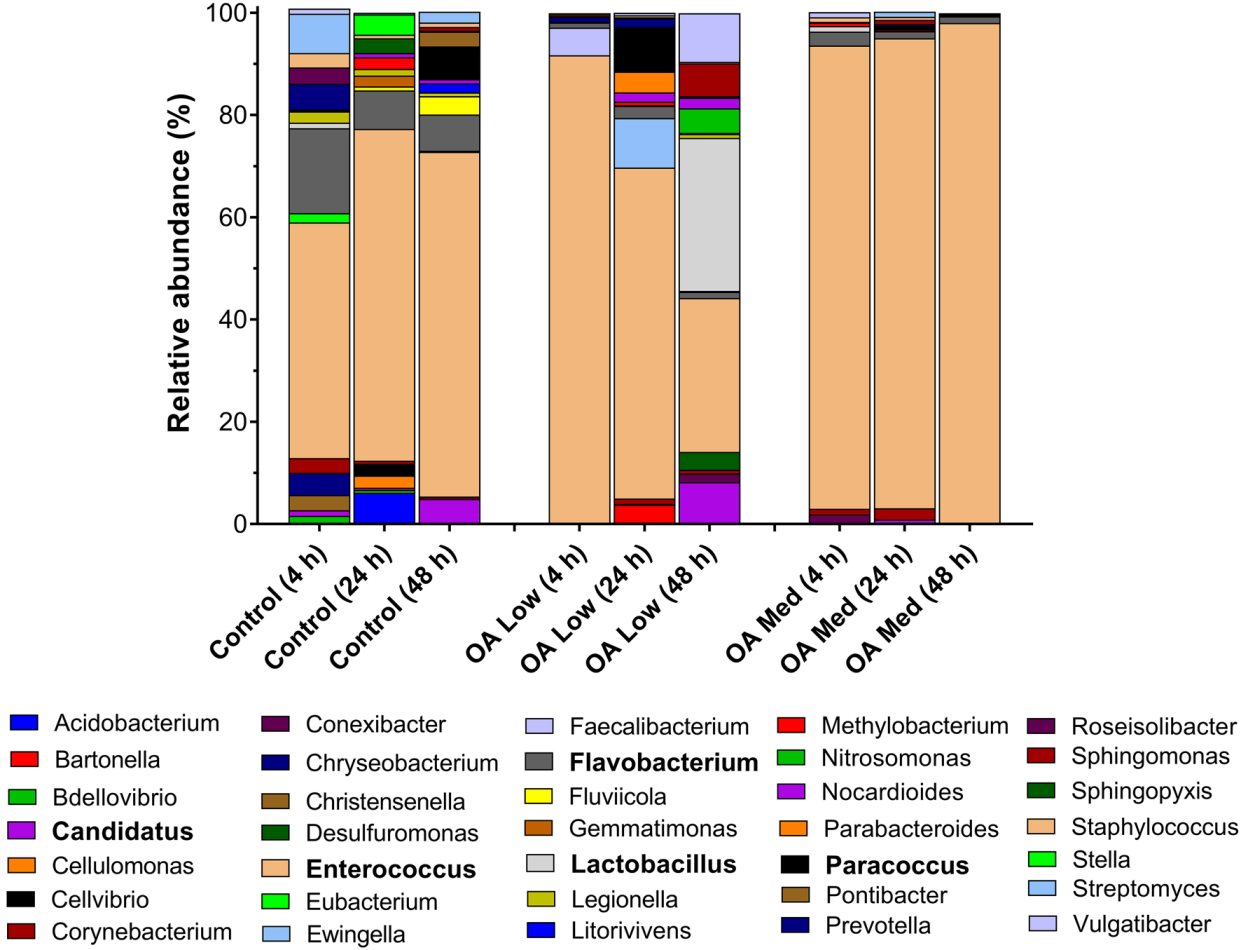
Genus-level bacterial composition (16S rRNA V3–V4) of the gut tissues of *Galleria mellonella* during okadaic acid (C_44_H_68_O_13_) intoxication. Insects were force-fed PBS (+ 5% v/v DMSO) or okadaic acid (80 μg/kg [low], 240 μg/kg [med]) and incubated at 30 °C in the dark. At 4, 24, and 48 h post-intoxication, insects were dissected and the gut tissues were snap frozen in liquid nitrogen (*n* = 9 per treatment across time points). For the control (48 h), ‘*Candidatus*’ represents proportions of *C. Trichorickettsia* (0.89) and *C. Monilibacter* (0.11). For the OA Low (48 h), ‘*Candidatus*’ represents proportions of *C. Neoehrlichia* (0.35) and *C. Koribacter* (0.65)

- Histopathology
  Histology slides were prepared from insects force-fed PBS or okadaic acid, and single blind assessed using the same grading system from Emery et al. (2019). Midgut tissues examined from untreated and negative controls (PBS +5% DMSO) showed some variation in architecture but contained few clear signs of damage; all slides were assigned grade 1 (<2 discrete changes), apart from two PBS slides graded 2 (one at 4 and another at 48 hours; Fig. 4). Transverse and longitudinal sections along the midgut of PBS-treated larvae revealed an intact arrangement of epithelial cells, including columnar, goblet, and regenerative cells (Fig. 5A–H). Epithelial folds, involved in nutrient absorption, were identified easily (Fig. 5B, D), as were the brush borders (with peritrophic matrix), basement membrane, and underlying muscle layer (Fig. 5C, G, H). The midgut lumen appeared mostly free of cellular debris (Fig. 5A, D, E), and there were few signs of immune cell (haemocyte) presence within the body cavity immediately surrounding the midgut (Fig. 5C).
  The low dose of OA (80 μg/kg) inflicted discrete/localised injury compared to the untreated and negative controls, which was apparent after 24–48 h (Fig. 4). The higher dose of OA (240 μg/kg) caused obvious damage to the larval midgut within the first 4 h of force feeding and became more extensive over the proceeding 48 h (Figs. 4, 6). Okadaic acid intoxication manifested as displacement of cells into the gut lumen, membrane blebbing/blistering, loss of cellular morphology, vacuolisation, and nuclear aberrations (pyknosis, karyolysis, and karyorrhexis) associated with cell death. Entire regions sloughed into the lumen by 4 and 24 h (Fig. 6A–D). In the body cavity, haemocytes were damaged and fragmented (Fig. 6E), which indicated the toxin made its way across the protective sublayers of the midgut. By 48 h, global tissue damage was apparent, and *in extremis*, the integrity of the entire midgut was compromised by necrosis and gross melanisation (Fig. 6G, H). Overall, okadaic acid jeopardised the insect alimentary canal with treatment (i.e., dose) and time accounting for ~ 79% and < 4%, respectively, of the variation within the data (Treatment, F_(3, 60)_ = 124.2, *P* < 0.0001; Time, F_(2, 60)_ = 8.2, *P* = 0.0007; Fig. 4).
- Resident bacteria (microbiome)
  Microbial signals in genomic DNA extracts of *G. mellonella* were interrogated by amplifying 16S (V3–V4) rRNA and sequencing using Illumina MiSeq. General diversity among the bacterial taxa colonising the gut tissues was low, whether intoxicated or naïve. In the absence of toxin, bacterial operational taxonomic units (OTUs) for the gut were dominated by four phyla in ascending order (Fig. 7): Actinobacteria (5–9%), Bacteroidetes (8–24%), Proteobacteria (20–23%), and Firmicutes (45–59%). Such abundance and composition are in good agreement with the previous studies (e.g., Dubovskiy et al. 2016). Oral administration of 80 μg/kg okadaic acid coincided with a transient shift in bacterial composition at 4 h post-exposure to ~90% Firmicutes and <1% Actinobacteria but seemed to return to (and exceed) pre-toxin diversity levels after 24–48 h. Conversely, using the higher dose of 240 μg/kg led to obvious reductions in all representative phyla (Acidobacteria and Spirochaetes were undetectable), except for Firmicutes, and corresponded to decreased Shannon (alpha) diversity and Chao-1 richness indices (3.7 ± 0.2 and 333 ± 21, respectively) compared to those fed 80 μg/kg (Shannon = 4.2 ± 0.34; Chao-1 = 438 ± 82) and 0 μg/kg (Shannon = 4.5 ± 0.1; Chao-1 = 453 ± 17) when considering all time points (Fig. 7). When probing down to genus level, *Enterococcus* represented ~46-67% (0 μg/kg toxin), ~30-91% (80 μg/kg toxin), and 91–98% (240 μg/kg toxin) of the top 35 identifiable taxa (Fig. 8), which does complement previous observations of enterococci prevalence across diverse *G. mellonella* tissue types (e.g., skin, gut, fat body, haemolymph; Krams et al. 2017; Allonsius et al. 2019). Interestingly, after 48-h exposure to a low dose of okadaic acid (80 μg/kg), larvae displayed ~30% each *Enterococcus* and *Lactobacillus*—perhaps the population was disrupted to such an extent that minor taxa could gain a foothold (both genera represent the Firmicutes)*.*
  Group-wise (community-level) comparisons on bacterial composition were analysed using PERMANOVA (999 permutations based on the Bray–Curtis method). Overall, the higher toxin dose (240 μg/kg) had a significant effect on the microbial composition when compared to the control larvae (0 μg/kg; *P* = 0.003, *R*^2^ = 0.172), however, was non-significant when compared to the lower dose (80 μg/kg; *P* = 0.3, *R*^2^ = 0.0911; Supplementary Table 2; Supplementary Fig. 1).

## Discussion

We assessed the putative impact of okadaic acid—one of the most common marine toxins and shellfish contaminants globally—on the integrity of gastric tissues and their resident microbiota using an alternative bioassay (*G. mellonella* larvae). In doing so, we accrued clear evidence that okadaic acid exposure at half (80 μg/kg) the upper regulated limit of 160 μg/kg enhanced susceptibility to infection by > 15% (Fig. 3a), and this acute event, coincided with visible damage to the gut epithelium and transient reductions in bacterial taxonomic diversity and richness (Figs. 4, 7). Increasing the acute dose to 240 μg/kg—approaching the published LD_50_ values for rodents (e.g., Aune et al. 2007, 2012)—caused extensive tissue disintegration, alongside prolonged bacterial dysbiosis (Figs. 6, 7). The severity of tissue damage was such that haemocytes (immune cells) circulating in the haemocoel (body cavity) surrounding the midgut were impaired (Fig. 6E)—indicating the toxin leaked across the no-longer-intact epithelium and underlying muscle. Immune-cytotoxic properties of okadaic acid at similar concentrations have been reported previously, including our work on insect haemocytes (Coates et al. 2019). DSP toxins are known to promote paracellular permeability in vitro (e.g., epithelial T84 cell monolayers; Tripuraneni et al. 1997) and okadaic acid can make its way from the luminal space to the blood in vivo (e.g., rodents; Ehlers et al. 2011). Epithelial sloughing, displacement into the lumen, vacuolisation, and cell death of the intoxicated larval gut are pathological features shared among many studies conducted in rodents. For example, intragastric intubation of okadaic acid at similar doses provoked villus fragmentation, shedding of intestinal and colonic epithelial cells, lesions, apoptosis and necrosis in the liver, kidney and forestomach in Nude and Wistar rats, and CD-1 mice (Berven et al. 2001; Liu et al. 2020Vieira et al. 2018). There is, however, variation in the extent of toxin sensitivity and pathological states among rodent models/species/strains of DSP, which may extend to different sources of *G. mellonella* (Coates et al. 2019 and references cited therein). Nevertheless, wax moth larvae provide a convenient platform for tracking broad histopathological changes in the absence/presence of biotic/abiotic factors as their size permits simultaneous assessment of multiple tissues/organs (Perdoni et al. 2014; Emery et al. 2019).

Most of the risk assessments available for marine toxins like okadaic acid are based on single, acute events performed on mice or in vitro using mammalian cell lines with a view to determining broad toxicological endpoints, such as, LD_50,_ lowest-observed-adverse-effects values and toxicity equivalency factors (e.g., Abal et al. 2018). The effects of continuous exposure of humans to contaminated shellfish tissue at market acceptable quantities (i.e., < 160 μg/kg) are largely uncharacterised. Some recent efforts have focussed on subacute or chronic low-dose exposure to determine longer term impacts on human health, and to a lesser extent, the shellfish hosts in situ. Liu et al. (2020) fed rats 80 μg/kg okadaic acid for 30 days, noting significant changes in colonic microbial loads within 3 days, in addition to local erosion of epithelial cells. Intraspecific variation in microbial abundance among intoxicated rats increased over the 30 days. Microbial diversity fluctuated, but there was a persistent inverse relationship between Firmicutes and Bacteroides abundances—signatures shared with our data. Daily intraperitoneal injections of 0.2–10 μg/kg for 120 days affected negatively the liver hepatocytes of mice (distorted endoplasmic reticulum, compromised mitochondrial membrane integrity, and apoptosis), as well as elevated levels of malondialdehyde (lipid peroxidation product) in serum (Wang et al. 2021). Proteomic assessment of the intoxicated mice retrieved 46 differentially expressed proteins with functions spanning metabolism, stress/chaperone factors, apoptosis, and the cytoskeleton. Although okadaic acid-induced DNA damage, modulation of DNA repair mechanisms, and links to tumour formation have been reported widely, they vary according to cell-line and/or host strain (Souid-Mensi et al. 2008; Valdiglesias et al. 2010, 2013). Long-term, pernicious effects of okadaic acid and DSP toxins are a considerable risk factor for gastrointestinal, hepatic, and pancreatic cancer according to several studies that co-correlated harmful algal bloom events over several decades with tumorigenesis along the alimentary canal (Cordier et al. 2000; Lopez-Rodas et al. 2006; Manerio et al. 2008; Del Campo et al. 2013).

Despite the increasing popularity of *G. mellonella* for general screening of microbes, toxins, and food additives (Champion et al. 2016; Maguire et al. 2016; Pereira et al. 2020; Emery et al. 2021), most experimental approaches inoculate directly into the body cavity (intrahaemocoelic injection)—circumventing natural routes of exposure for the GI tract—and relatively few studies monitor the microbiome. There is consensus, however, that enterococci are the most abundant and prevalent bacterial resident in the gut (Fig. 8; Bucher 1963; Jarosz 1979; Johnston and Rolff 2015; Dubovskiy et al. 2016; Krams et al. 2017; Ignasiak and Maxwell 2018; Allonsius et al. 2019; Polenogova et al. 2019; Kryukov et al. 2021). *Enterococcus* species, notably *E. mundtii*, are considered heritable symbionts that assist in managing the microbiota during metamorphosis and disabling them through targeted antibiotic treatment can make way for pathobionts to emerge (e.g., *Serratia;* Bucher 1963; Johnston and Rolff 2015). The low bacterial diversity/richness indices in *G. mellonella* can be attributed to their diet—based on wax and honey, which are replete in natural antimicrobials—and the bacteriocin-producing enterococci (Jarosz 1970; Johnston and Rolff 2015). Interestingly, Kong et al. (2019) demonstrated that long-chain hydrocarbon beeswax—the main dietary component of larvae—can be metabolised in the absence of microbes.

Infection of *G. mellonella* larvae via an oral route with bacteria (*Bacillus thuringiensis*; Dubovskiy et al. 2016), fungi (*Cordyceps militaris*; Kryukov et al. 2020), or parasite envenomation (*Habrobracon hebetor*; Polenogova et al. 2019) each promote dysbiosis—characterised by a common shift in Firmicutes relative abundances to either Proteobacteria- or Bacteroidetes-dominance, and reductions in diversity/richness indices. Again, our findings complement these reports (Fig. 7). Such dysbiotic events tend to enable pathogenic or opportunistic residents to proliferate and compromise the host—increasing their vulnerability to disease (e.g., spontaneous bacteriosis). We do observe changes in the *G. mellonella* gut microbiome and tissue architecture in the presence of okadaic acid, which can be linked to enhanced susceptibility to infection with a non-pathogen strain of *E. coli* (K12; Fig. 3). Antibiotics, such as oxytetracycline, can be used to disturb or purge gnotobiotic *G. mellonella* of the microbiota (Ignasiak and Maxwell 2018), and alongside their capacity to act as a host for many bacterial causes of gastroenteritis (e.g., *Helicobacter pylori*; Giannouli et al. 2014; *Vibrio parahaemolyticus*; Wagley et al. 2018, enteroaggregative *Escherichia coli*, Guerrieri et al. 2019), their use could extend to modelling/trialling novel antibiotic therapies against gut pathogens and scoping collateral consequences on the resident microbes and tissues.

## Concluding remarks

Doses of okadaic acid exceeding 160 μg/kg cause obvious gut erosion, cellular distortion, displacement, and death along the alimentary canal in both rodent and insect models and are linked to dysbiosis. Whether okadaic acid influences the microbiota directly or such changes are indirect due to a decline in pathological condition (i.e., gastropathy) remains uncertain. Herein, we have verified that *G. mellonella* larvae represent a reliable model for studying okadaic acid-induced gastrotoxicity at the whole organism, tissue, and cellular levels. More broadly, we demonstrate that a sub-lethal dose of okadaic acid (80 μg/kg) is potent enough to enhance susceptibility to infection from a routine laboratory bacterium. This suggests that humans exposed repeatedly to sub-regulatory levels of okadaic acid are at an increased risk of bacteriosis—likely from an existing resident gut symbiont or pathobiont.

## Supplementary Information

Below is the link to the electronic supplementary material.

Supplementary file1 (DOCX 174 KB)
