# Supplementary material for: The diarrhetic shellfish-poisoning toxin, okadaic acid, provokes gastropathy, dysbiosis and susceptibility to bacterial infection in a non-rodent bioassay, Galleria mellonella
Source: Arch Toxicol. 2021 Aug 10;95(10):3361–76. doi: 10.1007/s00204-021-03132-x (PMC8448676; doi:10.1007/s00204-021-03132-x)
Supplement: Supplementary file 1 — Supplementary file1 (DOCX 174 KB) [file 204_2021_3132_MOESM1_ESM.docx]

**Supplementary Table 1.** Survival analyses of *Galleria mellonella* larvae inoculated with okadaic acid only

|  | *P*BS | OA (80 μg/kg) | OA (240 μg/kg) | OA (400 μg/kg) |
| --- | --- | --- | --- | --- |
| Untreated | *X*^2^ (1) = 2.034  *P* = 0.1538 | *X*^2^ (1) = 3.105  *P* = 0.0780 | ***X*^2^ (1) = 25.76,**  ***P* < 0.0001** | ***X*^2^ (1) = 50.39,**  ***P* < 0.0001** |
| PBS |  | *X*^2^ (1) = 0.2319  *P* = 0.6302 | ***X*^2^ (1) = 20.3**  ***P* < 0.0001** | ***X*^2^ (1) = 47.06**  ***P* < 0.0001** |
| OA (80 μg/kg) |  |  | ***X*^2^ (1) = 17.42**  ***P* < 0.0001** | ***X*^2^ (1) = 43.72**  ***P* < 0.0001** |
| OA (240 μg/kg) |  |  |  | ***X*^2^ (1) = 10.69**  ***P* = 0.0011** |


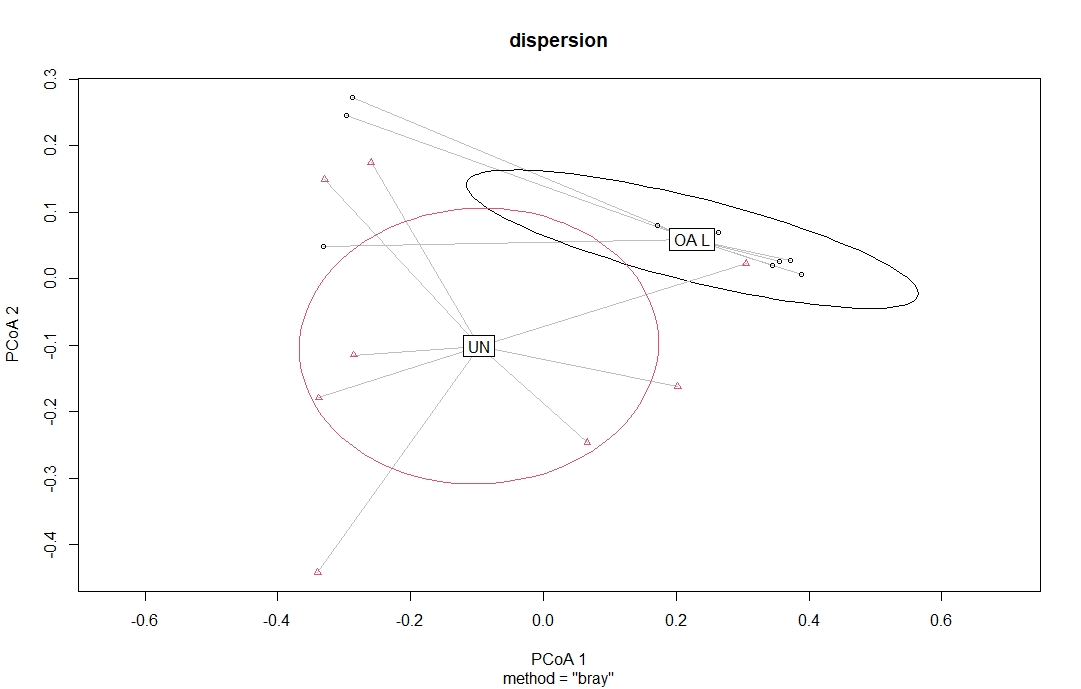


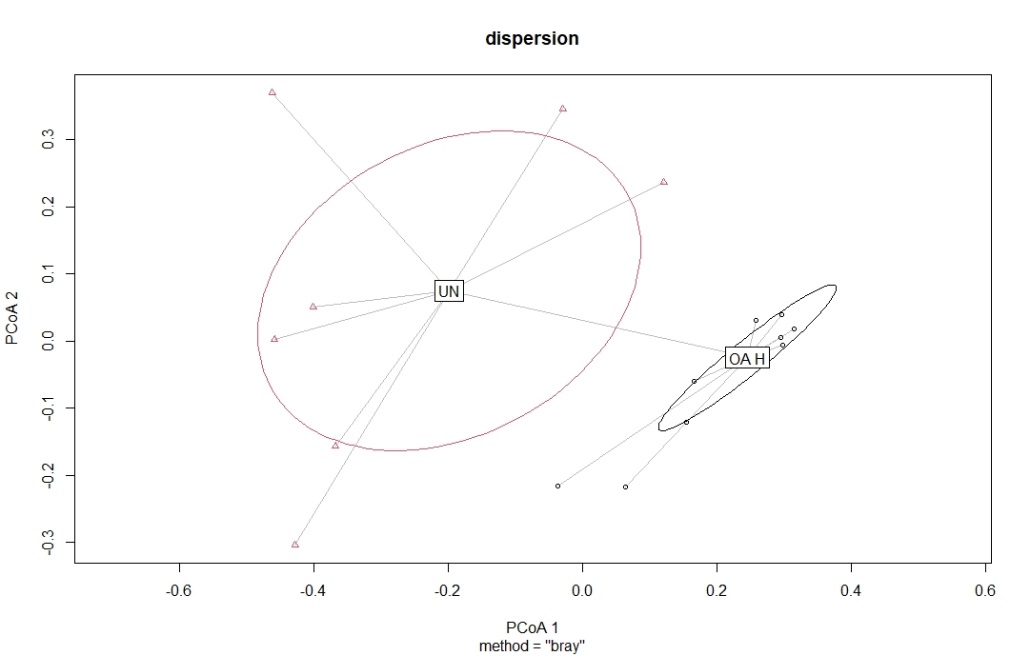


**Supplementary Figure 1.** Dispersion. Untreated insects versus those intoxicated with either the low dose of okadaic acid (80 μg/kg upper panel), or the high dose (lower panel, 240 μg/kg)

**Supplementary Table 2**. Results of permutation multivariate analysis of variance (PERMANOVA, *adonis* function) of OTUs retrieved from 16S rRNA data. Tests are based on the Bray-Curtis dissimilarity distances and 999 permutations.

1. untreated (0 μg/kg) insects versus intoxicated (low-dose, 80 μg/kg) insects

| **Source** | **DF** | **Sums Sq** | **F model** | **R2** | **Pr (>F)** |
| --- | --- | --- | --- | --- | --- |
| Treatment | 1 | 0.5794 | 1.5033 | 0.09109 | 0.067 |
| Residuals | 15 | 5.7810 |  | 0.90891 |  |
| Total | 16 | 6.3604 |  | 1 |  |

1. untreated (0 μg/kg) insects versus intoxicated (high-dose, 240 μg/kg) insects

| **Source** | **DF** | **Sums Sq** | **F model** | **R2** | **Pr (>F)** |
| --- | --- | --- | --- | --- | --- |
| Treatment | 1 | 0.9289 | 3.1096 | 0.1717 | **0.003** |
| Residuals | 15 | 4.4810 |  | 0.8289 |  |
| Total | 16 | 5.4099 |  | 1 |  |

1. low dose (80 μg/kg) versus high dose (240 μg/kg) of toxin

| **Source** | **DF** | **Sums Sq** | **F model** | **R2** | **Pr (>F)** |
| --- | --- | --- | --- | --- | --- |
| Treatment | 1 | 0.033 | 1.2136 | 0.0705 | 0.18 |
| Residuals | 16 | 3.9988 |  | 0.9295 |  |
| Total | 17 | 4.3021 |  | 1 |  |
